# Accuracy of semi-automated versus manual localisation of liver tumours in CT-guided ablation procedures

**DOI:** 10.1007/s00330-018-5498-8

**Published:** 2018-05-25

**Authors:** Hassan Boulkhrif, Ha Manh Luu, Theo van Walsum, Adriaan Moelker

**Affiliations:** 1 Department of Radiology and Nuclear Medicine, Erasmus MC, University Medical Center Rotterdam, Wytemaweg 80, 3015 CN Rotterdam, The Netherlands; 2 Department of Medical Informatics, Room Na 2506 Erasmus MC, Wytemaweg 80, Rotterdam, 3015 CN The Netherlands; 3 Avitech and FET, University of Engineering and Technology, Vietnam National University Hanoi, Hanoi, Vietnam

**Keywords:** Tomography, X-ray computed, Liver, Neoplasm, Ablation techniques

## Abstract

**Objectives:**

To compare the accuracy of liver tumour localisation in intraprocedural computed tomography (CT) images of computer-based rigid registration or non-rigid registration versus mental registration performed by interventional radiologists.

**Methods:**

Retrospectively (2009-2017), 35 contrast-enhanced CT (CECT) images incorporating 56 tumours, acquired during CT-guided ablation procedures and their corresponding pre-procedural diagnostic CECTs were retrieved from the picture archiving and communication system (PACS). The original intraprocedural CECTs were de-enhanced to create a virtually unenhanced CT image (VUCT). Alignment of diagnostic CECTs to their corresponding intraprocedural VUCTs was performed with non-rigid or rigid registration. Mental registration was performed by four interventional radiologists. The original intraprocedural CECT served as the reference standard. Accuracy of tumour localisation was assessed with the target registration error (TRE). Statistical differences were analysed with the Wilcoxon signed-rank test.

**Results:**

Non-rigid registration failed to register two CT datasets, incorporating four tumours. In the remaining 33 datasets, non-rigid, rigid and mental registration showed a median TRE of 3.9 mm, 9.0 mm and 10.9 mm, respectively. Non-rigid registration was significantly more accurate in tumour centre localisation in comparison to rigid (*p* < 0.001) or mental registration (*p* < 0.001). Rigid registration was not statistically different from mental registration (*p* = 0.169). Non-rigid registration was most accurate in localising tumour centres in 42 out of 52 tumours (80.8%), while rigid and mental registration were most accurate in only seven (13.5%) and three (5.8%) tumours, respectively.

**Conclusions:**

Computer-based non-rigid registration is statistically significantly more accurate in localising liver tumours in intraprocedural unenhanced CT images in comparison to rigid registration or interventional radiologists’ mental mapping abilities.

**Key Points:**

*• Computer-based non-rigid registration is better (p < 0.001) in localising target tumours prior to ablation in intraprocedural CT images in comparison to rigid registration or interventional radiologists’ mental mapping abilities.*

*• Human experts perform sub-optimal localisation of target tumours when relying solely on mental mapping during challenging CT-guided procedures.*

*• This non-rigid registration method shows promising results as a safe alternative to intravenous contrast media in liver tumour localisation prior to ablation during CT-guided procedures.*

## Introduction

Radiofrequency ablation (RFA) and microwave ablation (MWA) are widely accepted minimally invasive procedures for the treatment of malignant liver tumours [1, 2]. To localise liver tumours during image-guided ablation procedures, ultrasound (US) is often preferred as the initial imaging modality. Computed tomography (CT) is used to target tumours that cannot be localised with US. Interventional radiologists mentally map tumour information such as location and size from the diagnostic pre-procedural contrast-enhanced CT (CECT) scan and apply it to the intraprocedural unenhanced CT scan. This is challenging, since few landmarks are available in unenhanced CT scans [3]. Diagnostic and intraprocedural CT scans are generally acquired weeks apart and, hence, tumour evolution, image quality, patient positioning and respiratory motion may hamper localisation [4, 5].

Several approaches have been developed to reduce the need for contrast agent during challenging ablation procedures. Van Tilborg et al. [6] have successfully placed catheters in the hepatic and superior mesenteric arteries, through which small volumes of contrast agent were administered intermittently for real-time liver tumour visibility during CT-guided ablations. Other groups have pursued a contrast-agent free approach for liver tumour localisation based on image registration, in which diagnostic magnetic resonance imaging (MRI) or CT images were brought into anatomical alignment with their corresponding intraprocedural CT images [4, 7]. Registration accuracy was assessed in those studies, mostly focusing on liver borders or clearly visible anatomical landmarks. However, no accuracy of localising tumours prior to ablation was investigated in comparison with mental registration abilities of human experts. In other words, the benefit of computed registration methods compared to mental registration by the interventional radiologist is largely unknown.

This study therefore aimed to determine the accuracy of localising tumours with computer-based rigid or non-rigid registration methods in comparison to mental registration performed by interventional radiologists during CT-guided percutaneous ablation of liver tumours.

## Materials and methods

### Image data acquisition

Clinical datasets were obtained retrospectively from 35 subjects (incorporating 56 tumours) treated for malignant hypovascular and hypervascular liver tumours with CT-guided percutaneous ablation procedures from June 2009 to April 2017. A subject was eligible for inclusion when both the diagnostic and its corresponding intraprocedural CECT was available. Only clearly visible liver tumours in both CT datasets were included in the experiment. The institutional review board (IRB) of the Erasmus MC approved that the Medical Research Involving Human Subjects Act does not apply to this study and that no informed consent was required for anonymised processing according to the local directives for retrospective studies (MEC-2014-385). All data were anonymised prior to processing.

### Image processing: creating virtual de-enhanced intraprocedural CT images

**Fig. 1:**
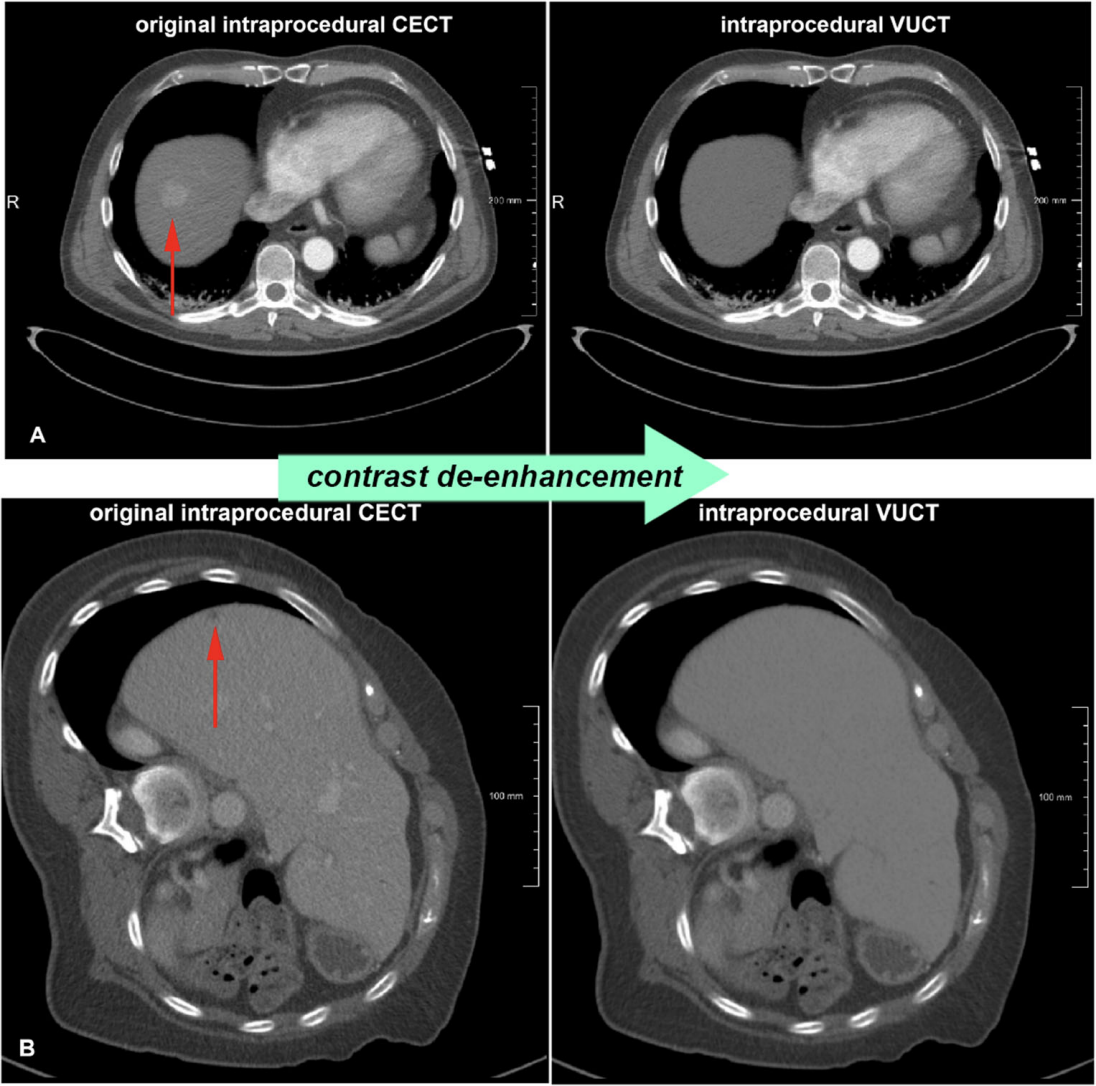
Examples of intraprocedural VUCTs based on contrast de-enhancement in the livers of intraprocedural CECTs. **a** An example of the virtual de-enhancement of the liver incorporating a hypervascular tumour (HCC). **b** An example of a hypovascular tumour (colorectal metastasis). Note that the contrast-enhanced vessels are also unenhanced. *VUCT* virtual unenhanced CT image, *CECT* contrast-enhanced CT image, *HCC* hepatocellular carcinoma

Intraprocedural CECTs acquired during interventions were retrospectively contrast de-enhanced using an in-house developed tool that was built in the medical imaging research framework MeVisLab 2.7.1 (MeVis Medical Solutions, Bremen, Germany; www.mevislab.de). The purpose of this contrast de-enhancement of the liver was to simulate an intraprocedural unenhanced CT image (Fig. 1). The intraprocedural virtually unenhanced CTs (VUCTs) were created by replacing hyper-attenuated regions [hepatocellular carcinomas (HCCs) and contrast-enhanced vessels] or manual segmented hypo-attenuated regions (metastases) with values that are representative for the normal liver tissue. Hilar vessel silhouettes and very high intensity pixels (such as surgical clips and calcifications) were excluded from the replacement to normal liver tissue values. By creating intraprocedural VUCTs based on the original intraprocedural CECTs, we established a non-invasive distinct ground truth for the tumour’s location.

### Image registration: background information

**Fig. 2:**
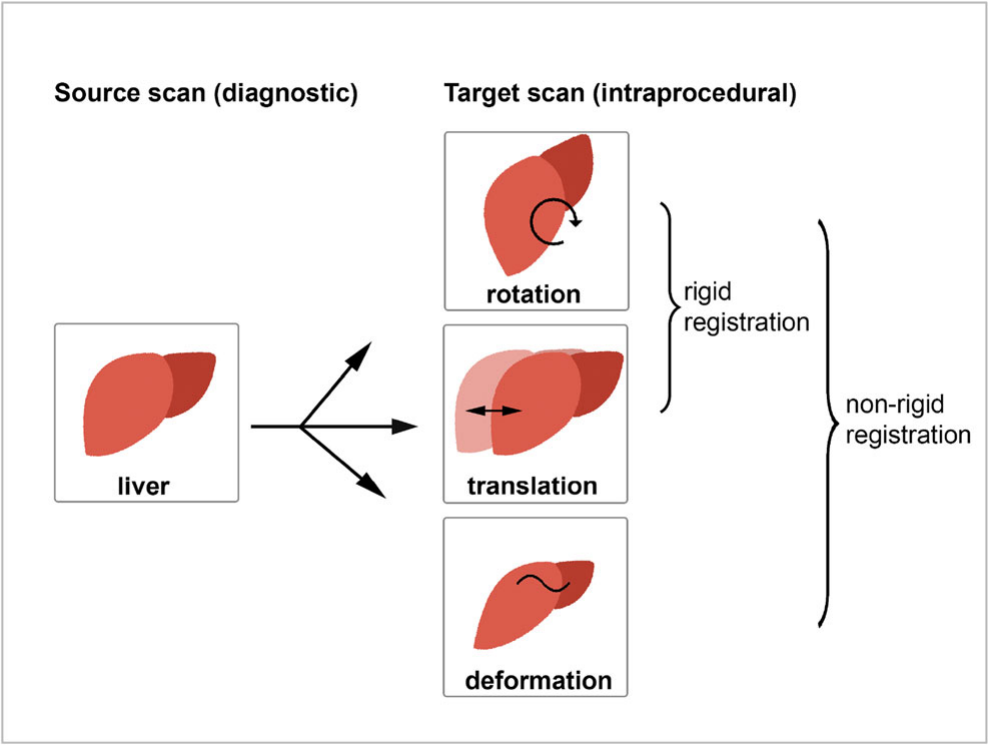
Anatomical alignment between source and target scans of the liver with rigid and non-rigid registration

Image registration software uses algorithms for the anatomical alignment of datasets originating from several imaging modalities or from the same modality but acquired at different moments [5]. If alignment is performed adequately, anatomical information from the diagnostic scan (source) can be projected in the non-enhanced CT image (target), allowing accurate localisation of the tumour without the use of intraprocedural contrast media. Computer-based image registration can be performed rigidly or non-rigidly (elastic). Rigid registration only corrects rotation and translation in the anatomical matching of two datasets, whereas non-rigid registration is more flexible and also permits deformation. The latter is especially of interest in ablation procedures involving the liver, in which respiratory motion or intraprocedural rotation of the patient can lead to liver deformation (Fig. 2) [8].

### Image registration: localisation experiment

**Fig. 3:**
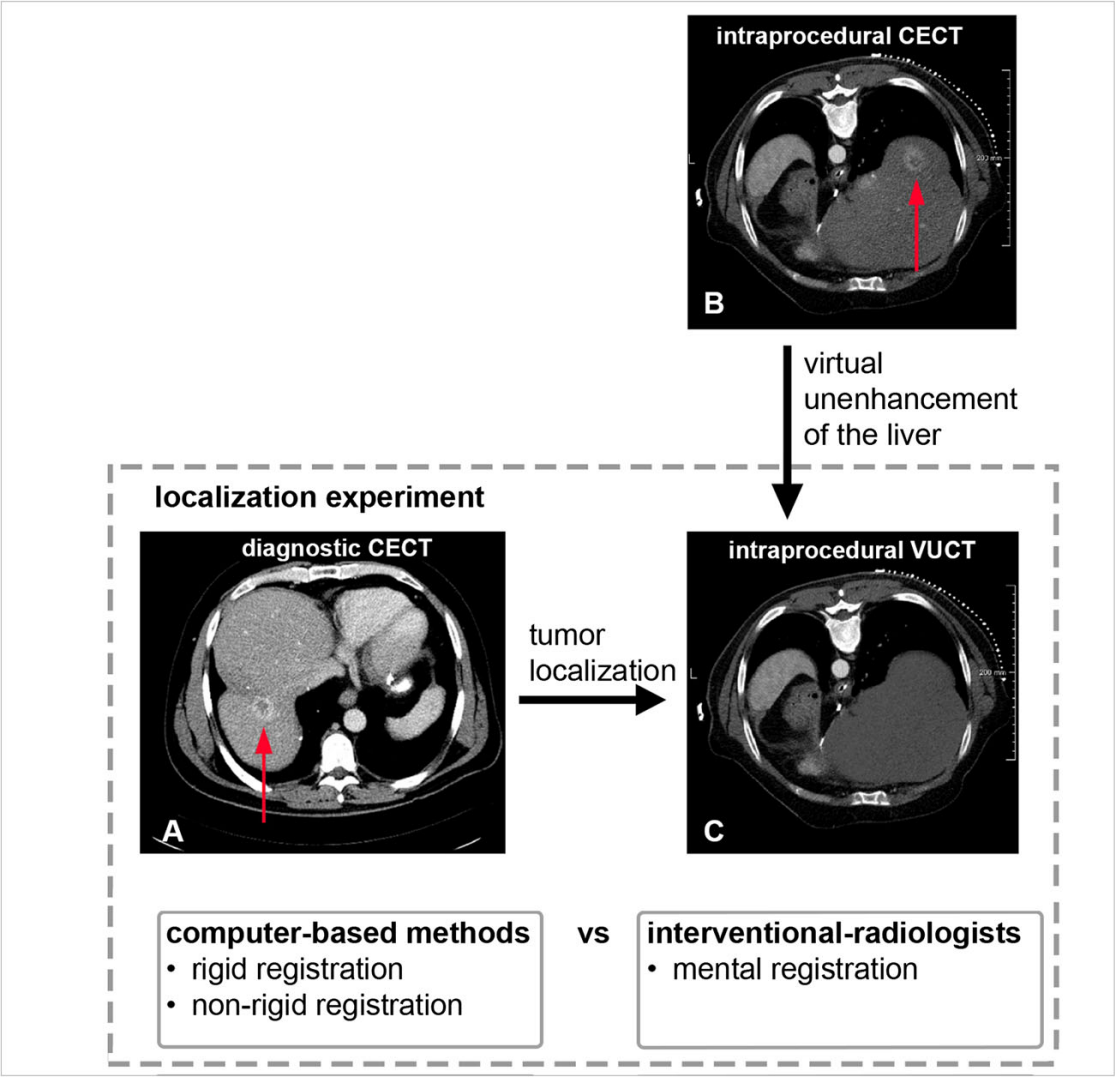
Localisation experiment: the axial diagnostic CECT (**a**) was used by the interventional radiologists and the computer-based registration methods to localise the target tumour centre in the axial intraprocedural VUCT (**c**). The original intraprocedural CECT (**b**) served as the reference standard for the ground truth location of the tumour in the intraprocedural VUCT. Note that the patient was extremely rotated in this intraprocedural CT scan. *CECT* contrast-enhanced CT image, *VUCT* virtual unenhanced CT image

In the localisation experiment (Fig. 3), liver tumour centres were annotated in the intraprocedural VUCTs by using corresponding diagnostic CECTs. Tumour centre localisation by semi-automated non-rigid or rigid registration methods was compared to the mental registration abilities of interventional radiologists.

#### Semi-automated rigid and non-rigid registration methods

**Fig. 4:**
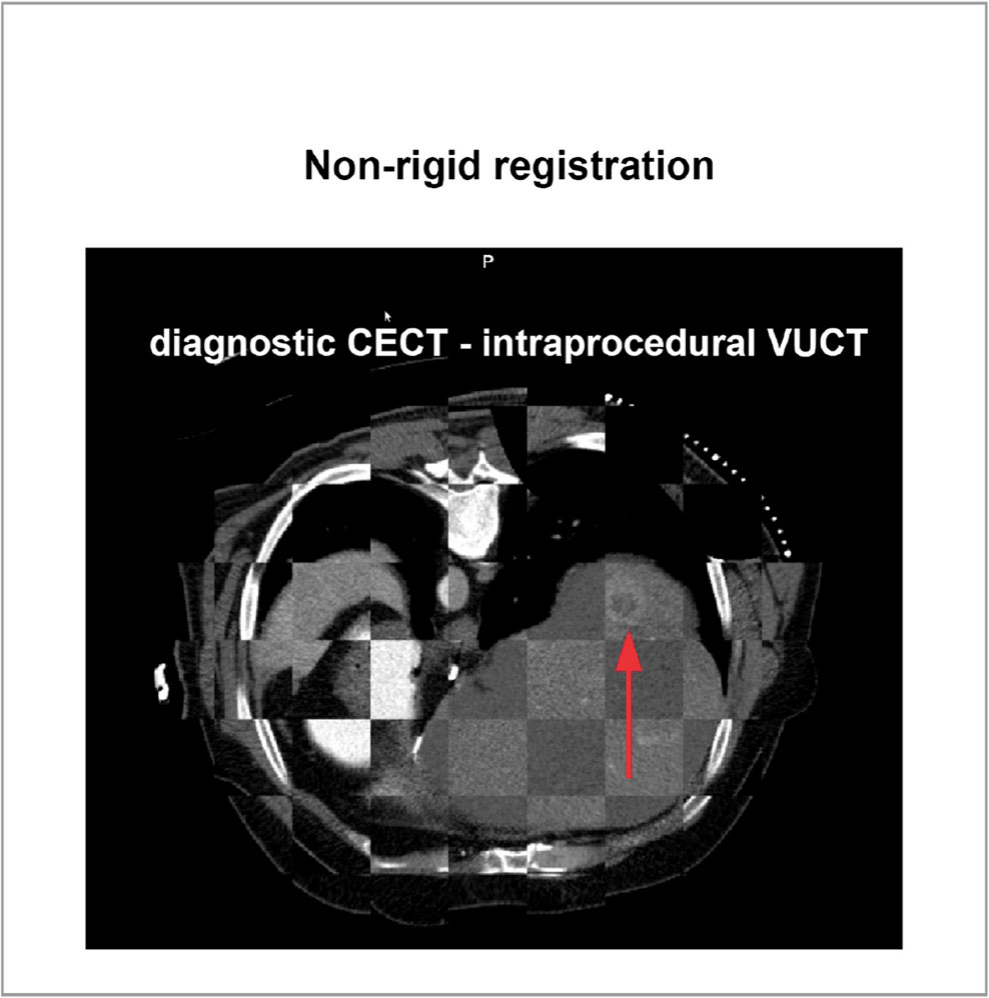
Non-rigid registration of a diagnostic CECT with its corresponding VUCT. The target tumour from the diagnostic CECT was anatomically matched into the intraprocedural VUCT (*red arrow*). Note that spatial alignment was restricted to the liver regions. *CECT* contrast-enhanced CT image, *VUCT* virtual unenhanced CT image

Rigid and non-rigid registration methods were investigated in this study [7, 9]. First, a mask of the liver was roughly outlined in the diagnostic CECT. To correct for rotation and translation differences, an initial rigid alignment between the diagnostic CECT and intraprocedural VUCT was pursued. The non-rigid registration method used B-spline modelling to compute the deformation of the liver between the diagnostic CECT and the intraprocedural VUCT. After registration, the outlined tumour from the diagnostic CECT was transformed to the intraprocedural VUCT and subsequently the centre of the tumour was computed. Elastix (elastix.isi.uu.nl), an open-source platform, was used to perform the registration [10]. Figure 4 shows an example of non-rigid registration of a diagnostic CECT to its intraprocedural VUCT, in which the clearly visible target tumour from the diagnostic CECT is anatomically matched into its corresponding intraprocedural VUCT.

#### Mental registration approach

Mental registration was performed by four interventional radiologists. The interventional radiologists were presented the diagnostic CECT and its corresponding intraprocedural VUCT in which the tumour was inconspicuous. Subsequently, they were asked to manually pinpoint the centre of the tumour in the intraprocedural VUCT by using their mental mapping abilities. The annotations of four interventional radiologists per tumour were averaged for the assessment of the mental registration approach.

### Reference standard

The original intraprocedural CECTs served as the distinct ground truth location for the liver tumour centres. An experienced abdominal radiologist (>5 years of experience) annotated the tumour centres in the intraprocedural VUCTs by using the corresponding original intraprocedural CECTs. These manual annotations served as the reference standard for the mental registration approach. Computer-based registration methods are potentially able to annotate in between CT slices, in contrast to mental registration which can annotate in-plane only. To prevent in-between slice errors, tumour centres were calculated by slice-by-slice segmentation of the tumour in the original intraprocedural CECT and subsequent calculation of the centre of gravity of the volumetric tumour shape. These calculated tumour centres were used in the evaluation of the computer-based methods.

### Assessment of accuracy

The target registration error (TRE) was used to assess the registration accuracy. The TRE is the Euclidean distance between the annotated tumour centre in the intraprocedural VUCT and the ground truth tumour centre in the original intraprocedural CECT [11]. A perfect tumour match will result in a TRE of 0 mm. A larger TRE correlates with suboptimal tumour localisation.

### Analysis

Normal data distribution was assessed with the Shapiro-Wilk test. Statistical differences were analysed with IBM SPSS 24.0 using the Wilcoxon signed-rank test to compare the differences in TRE between the three groups. To adjust for multiple (*n* = 3) comparisons, we performed a Bonferroni correction and differences were considered to be significant when the *p* value was less than 0.016 (= 0.05/3).

## Results

All primary liver malignancies (*n* = 35) were HCCs and showed a hypervascular appearance on the original intraprocedural CECTs. Secondary tumours showed a hypovascular appearance on the original intraprocedural CECTs and mainly concerned colorectal metastases (*n* = 16) or metastases from neuroendocrine (*n* = 3), gastric (*n* = 1) or breast (*n* = 1) origin. All 56 included tumours were more or less round and symmetrical shaped with an average size of 20.4 mm ± 9.4 (range, 6.1-60.0 mm; median, 18.3 mm). With regard to subject rotation, 12 out of 35 subjects were scanned in a rotated position during the original ablation procedure for practical reasons such as trajectory planning determined by the interventional radiologist. These patients were positioned in the right position before acquiring the intraprocedural CECT. Of those 12 rotated subjects, three were extremely rotated (48, 182, 200 degrees) and nine were rotated to a lesser extent (< 15 degrees). The average acquisition time between the diagnostic and its corresponding intraprocedural CT dataset was 59 days.

**Fig. 5:**
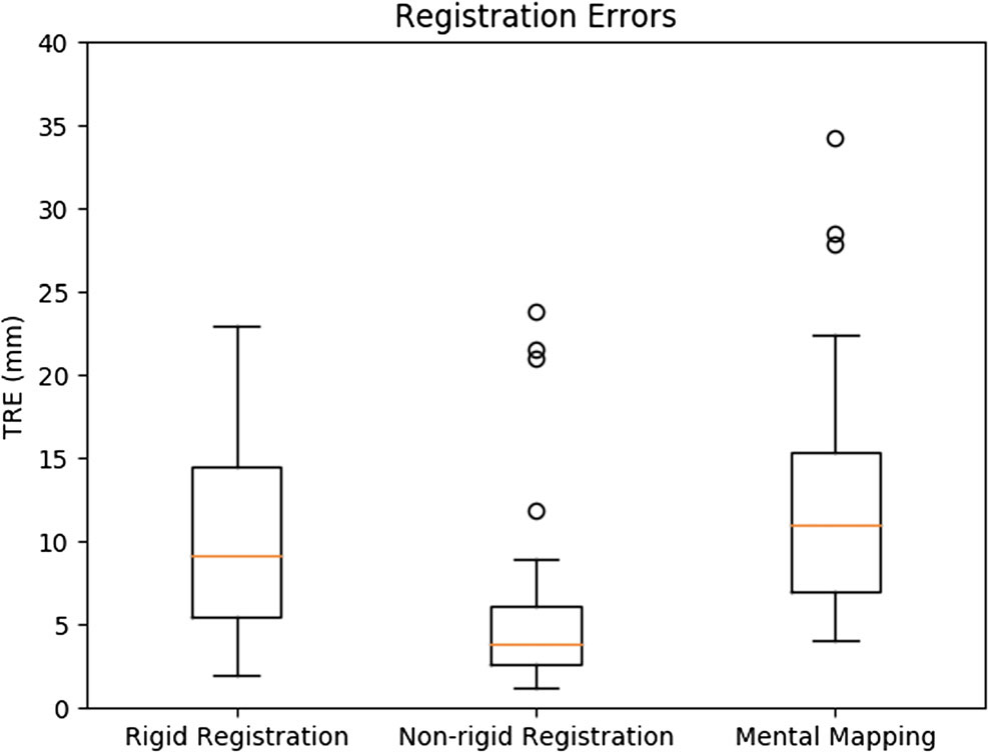
Registration accuracy based on TRE for non-rigid, rigid and mental registration, depicted in a boxplot. The median registration error is based on 52 tumours for non-rigid, rigid and mental registration. Non-rigid registration did not result in registration of tumours 3, 4, 35 and 36. These four tumours correspond with extreme outliers in the rigid registration method which are not depicted in this figure (tumour 3, 133.4 mm; tumour 4, 42.8 mm; tumour 35, 118.5 mm; tumour 36, 121.1 mm). *TRE* target registration error

Non-rigid, rigid and mental registration methods were applied to 35 pairs of CT datasets incorporating 56 tumours. The median TREs of the three methods are depicted in a box plot (Fig. 5). Non-rigid registration resulted in 52 completed annotations out of 56, because it was not able to find a suitable transformation for four tumours (tumour no. 3, 4, 35 and 36) originating from two CT datasets (the registration converges to a solution where the livers do not overlap). Rigid and mental registration were able to annotate all 56 tumour centres in the intraprocedural VUCT. Nonetheless, rigid registration resulted in extreme TRE outliers for those four specific tumours (tumour 3, 133.4 mm; tumour 4, 42.8 mm; tumour 35, 118.5 mm; tumour 36, 112.1 mm). Mental registration resulted in smaller TREs for tumours 3, 4, 35 and 36 (20.4 mm, 12.7 mm, 6.4 mm and 20.8 mm, respectively). As the aim was to compare the localisation accuracy between the registration methods, these four tumours were excluded from the comparison. Based on the analysis of the remaining 52 tumours, the non-rigid registration approach showed the smallest median TRE of 3.9 mm in comparison to rigid registration (9.0 mm) or mental registration (10.9 mm) (*see* Table 1).

**Table 1:** Comparison of registration methods (*n =* 52 tumours)

Registration approach	Median TRE (mm)	Mean TRE (mm)	Registration time (s)	Most accurate^a^ per tumour (%)
Mental registration	10.9	11.7 ± 6.6	108	3 out of 52 (5.8)
Rigid registration	9.0	10.1 ± 6.0	23	7 out of 52 (13.5)
Non-rigid registration	3.9	5.3 ± 4.8	105	42 out of 52 (80.8)

*TRE* target registration error

^a^Based on smallest TRE per tumour

Furthermore, non-rigid registration was most accurate in localising tumour centres (e.g. smallest TRE) in 42 out of 52 tumours (80.8%), while rigid and mental registration were most accurate in seven (13.5%) and three (5.8%) tumours, respectively.

The average registration time for non-rigid, rigid and mental registration was 1 min 45 s per subject, 23 s per subject and 1 min 48 s per tumour, respectively, as shown in Table 1.

As the TRE data were skewed and the Shapiro-Wilk test indicated non-normality (*p* < 0.05) for the three groups, a Wilcoxon signed-ranks test was performed. Differences were considered significant when the Bonferroni-corrected *p* value was less than 0.016. Non-rigid registration showed statistically significantly smaller TREs than rigid registration (*p* < 0.001) or mental registration (*p* < 0.001). Rigid registration on the other hand, did not show statistically significant differences in comparison to mental registration (*p* = 0.169).

## Discussion

Our results show that non-rigid registration yields better localisation accuracy than the less advanced rigid registration or the conventional approach of mental registration performed by interventional-radiologists.

Previous studies have focused on the spatial alignment of liver borders between diagnostic and intraprocedural images [4, 7]. However, it is difficult to compare our study, because they have focused on indirect parameters related to organ alignment instead of tumour alignment, which are surrogates of the TRE. Direct non-invasive evaluation of the TRE is only possible with the VUCT method presented, due to the availability of a distinct ground truth location of the tumour in the original intraprocedural CECT. Nonetheless, accuracies reported in other studies are similar to ours. Luu et al. [7] showed a mean accuracy of 5.3 mm based on the mean surface distance (MSD) and a dice similarity coefficient (DSC) of 91% for non-rigid registration between diagnostic and intraprocedural CT images. Elhawary et al. [4] non-rigidly registered livers from diagnostic MRI images to intraprocedural CT images and reported a mean TRE of 4.1 mm and a DSC of 97%.

This study demonstrates that adequate liver tumour localisation may be possible without contrast media prior to ablation in case of CT-guided procedures. We have shown that the described non-rigid registration method outperforms human experts and achieves a median registration accuracy of 3.9 mm. This accuracy is clinically acceptable when the safety margin for thermal ablation treatment is at least 5 mm [11]. Nonetheless, four non-rigidly registered tumours showed a TRE > 10 mm. A closer look at these intraprocedural CT images showed no satisfying explanation for these errors, e.g. no extreme rotation or aberrant anatomical features were present. More accurate results might be achieved with registration methods based on non-linear biomechanical modelling. These methods compute deformations that transform source to target image and allow even more accurate anatomical matching [12].

The computational time for the described non-rigid registration method is less than 2 min. This is comparable to the time required for mental registration of liver tumours. The only manual user input required for non-rigid registration is rough outlining of the liver in the diagnostic and intraprocedural CT images [7] and drawing of a single anteroposteral axis line from the spinous process to the sternum. Automated segmentation methods of liver contours may be used as a substitute [13].

### Future directions

Registration times in the range of milliseconds could enable real-time visualisation and tumour targeting. A possible approach for further reduction in time, and therefore real-time correction of respiratory and patient movement, is whole liver registration at the start of the procedure, followed by registration of serial slabs of 4-6 cm during the ablation procedure.

Tumour centres annotated with non-rigid registration are presented in *x*, *y* and *z* coordinates, which enables interventional robotics (e.g. needle-placement devices) to be coupled for further automation of the CT-guided ablation process [14–16]. This may allow interventional radiologists robotisation of ablation from an interventional cockpit.

### Limitations

A limitation is the use of intraprocedural VUCTs. The purpose of contrast de-enhancement was to mask structures which were revealed due to contrast use, while leaving gross liver anatomy intact. We acknowledge that manipulation of the original intraprocedural CECT could have resulted in alterations of granularity of the image. However, this was not mentioned as an issue for tumour localisation by the human observers. A second limitation is the retrospective nature of the study. Nevertheless, we think that important lessons can be learned from this study, such as the feasibility of accurate tumour localisation with computer-based non-rigid registration in CTs acquired during interventions and potentially improving tumour treatment while reducing the use of IV contrast media.

## Conclusions

Computer-based non-rigid image registration is more accurate in depicting the location of liver tumours in intraprocedural unenhanced CT images in comparison to rigid registration or interventional radiologists’ mental mapping in a retrospective study. Non-rigid registration shows promising results as an alternative to intravenous contrast media when the clinician experiences undetectable liver tumours during CT-guided ablation procedures.

## Abbreviations and acronyms

DSC: Dice similarity coefficient
HCC: Hepatocellular carcinoma
MSD: Mean surface distance
MWA: Microwave ablation
RFA: Radiofrequency ablation
TRE: Target registration error
VUCT: Virtual unenhanced CT image
